# Piezo1 channel: structure, mechanogating mechanism, functions, diseases and therapeutic strategy

**DOI:** 10.1186/s43556-026-00549-7

**Published:** 2026-08-19

**Authors:** Qixiang Wu, Ying Hu, Yuhan Wang, Fuqiang Zhu, Qiongyu Xu, Ling Xu, Jinguo Xu, Tao Xu

**Affiliations:** 1 Inflammation and Immune Mediated Diseases Laboratory of Anhui Province, School of Pharmaceutical Sciences, Anhui Medical University, No.81 Meishan Road, Hefei, Anhui 230032 P.R. China; 2 State Key Laboratory of Pharmaceutical Biotechnology, School of Life Sciences, Nanjing University, Nanjing, Jiangsu P.R. China; 3 International Cooperation and Exchange Department, Shanghai General Hospital, 85/86 Wujin Road, Hongkou District, Shanghai, 200434 P.R. China; 4 Department of Clinical Medicine, The Second School of Clinical Medical, Anhui Medical University, Hefei, P.R. China; 5 Department of Respiratory Medicine, The Anhui Chest Hospital, No.397 Jixi Road, Hefei, Anhui 230032 P.R. China; 6 Department of Cardiovascular Surgery, First Affiliated Hospital of Anhui Medical University, No. 218 Jixi Road, Shushan District, Hefei, Anhui 230022 P.R. China

**Keywords:** Piezo1, Mechanosensitive channel, Mechanogating mechanism, Pharmacological modulator

## Abstract

The Piezo1 channel is a mechanosensitive, non-selective cation channel that converts mechanical forces into electrochemical signals, playing pivotal roles in vertebrate physiology. Structurally, Piezo1 features a distinctive trimeric propeller structure that undergoes conformational changes in response to membrane tension, enabling mechanogating. Accordingly, Piezo1 is involved in a broad spectrum of physiological processes, including vascular development and homeostasis, bone and cartilage formation, skeletal muscle growth, neural development, sensory perception, immune regulation, and cellular volume regulation. Accumulating evidence indicates that mutations or dysregulation of Piezo1 are closely associated with a variety of human diseases, including genetic diseases, cardiovascular diseases, infectious diseases, autoimmune diseases, and cancer. Therefore, Piezo1 has emerged as a potential therapeutic target. Currently, the exploration of pharmacological modulators targeting Piezo1, as well as emerging approaches such as gene therapy, artificial intelligence (AI)-driven drug discovery, and advanced drug delivery systems, offer potential avenues for the development of Piezo1-targeted therapeutic strategies. However, these approaches still face significant challenges regarding specificity, in vivo targeting, and context-dependent effects. This review systematically summarizes the structure, mechanogating mechanisms, physiological and cellular functions of Piezo1, as well as its associations with human diseases. Based on this, the limitations of current Piezo1-targeted therapeutic strategies and their future developmental directions are highlighted, while the therapeutic potential of targeting Piezo1 is emphasized.

## Introduction

Mechanosensitive ion channels are a class of membrane proteins that can sense cell membrane tension or mechanical stimuli and convert them into ion transmembrane flow and electrochemical signals [1]. Among the numerous known mechanosensitive channels, the Piezo family is unique. They function as specialized channel proteins for sensing mechanical stimuli and mediating mechano-electrical transduction in mammalian cells. The Piezo family comprises two members, Piezo1 and Piezo2, which were first discovered by the team of Ardem Patapoutian in 2010 [2, 3]. This groundbreaking discovery filled a long-standing gap in our understanding of how mammalian cells sense physical environment and was later honored with the 2021 Nobel Prize in Physiology or Medicine.

Compared to Piezo2, which is primarily distributed in sensory neurons and responsible for tactile signals such as light touch and proprioception, Piezo1 is widely expressed across a diverse range of human cell types, including endothelial cells (ECs), erythrocytes, osteoblasts, chondrocytes, and various immune cells [4–6]. Consequently, Piezo1 has garnered increasing attention in studies of various critical physiological processes, including vascular development, skeletal system formation, immune regulation, and cell volume homeostasis [7–9]. In recent years, with the rapid development of high-resolution cryo-electron microscopy (cryo-EM), in situ conformational dynamics imaging, and pharmacological research, the understanding of its physiological functions and mechanotransduction mechanisms has deepened continuously [10, 11]. To date, accumulating evidence has demonstrated that Piezo1 dysfunction is closely associated with the onset and progression of various human diseases. As a result, targeting Piezo1 to modulate its mechanotransduction activity has emerged as a highly promising therapeutic strategy.

This review aims to provide a comprehensive overview of the latest advances in the field of Piezo1 biology. Firstly, the structure of the Piezo1 channel and its mechanosensitive gating mechanism are discussed. Subsequently, the physiological and cellular functions of Piezo1 are explored, followed by an examination of the implications of Piezo1 dysfunction in human diseases. Finally, targeted therapeutic strategies for Piezo1 are reviewed, with particular emphasis on the critical research gaps and major challenges that need to be addressed.

## Structure of the Piezo1 channel

**Fig. 1:**
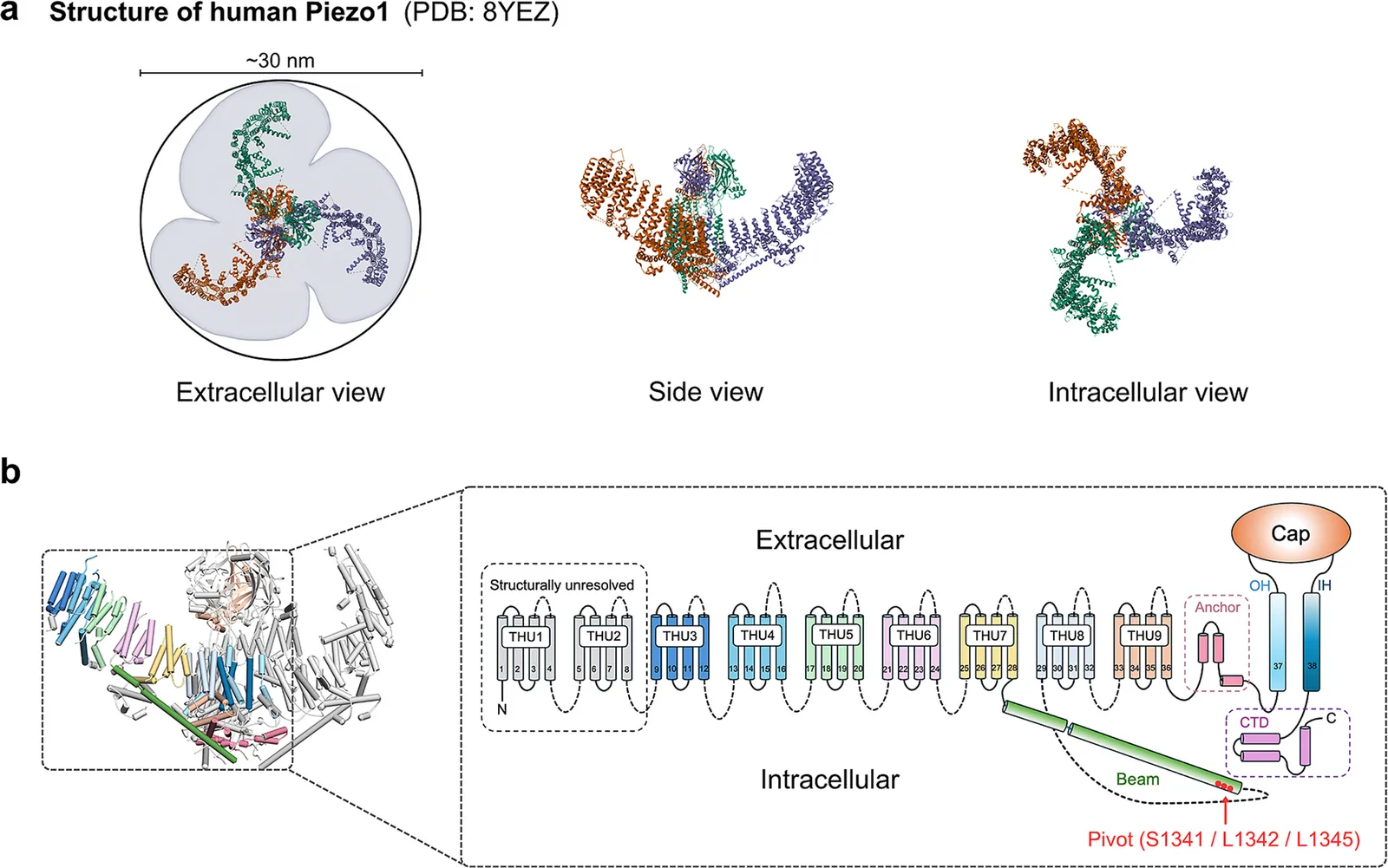
Structure and transmembrane topology of Piezo1 channel.** a** Structure of human Piezo1 (Image from the RCSB PDB (RCSB.org) of PDB ID 8YEZ, PDB DOI: https://doi.org/10.2210/pdb8YEZ/pdb). **b** 38-transmembrane topology model of a single Piezo1 subunit

Piezo1 is a large transmembrane protein evolutionarily conserved across animals and plants, though absent in bacteria and yeast [12, 13]. In humans, the Piezo1 gene resides on chromosome 16q24.3, comprising 51 exons that encode a protein of 2521 amino acids. Structurally [13], Piezo1 assembles as a trimeric channel resembling a three-bladed propeller, and its low-pass filtered cryo-EM density map reveals a disk structure with a diameter of approximately 30 nm in the extracellular view (Fig. 1a) [14]. Within this trimeric assembly, each protomer contains nine transmembrane helical units (THUs), with each THU composed of four transmembrane helices (TM helices), forming a total of 36 TM helices that constitute the peripheral blades region [14, 15]. The THUs in Piezo1 are interconnected by intracellular loops that are intrinsically disordered, among which only a few structured elements (such as the beam and clasp) have been resolved [16]. Although these intracellular loops have been largely overlooked, they contribute to the flexibility of the blade structure and are thought to be important for potential protein regulation and interactions [17, 18]. The central region of the trimeric assembly constitutes a functionally essential pore module, which is architecturally organized by two TM helices located near the protein center, known as the outer helix (OH) and inner helix (IH), along with an extracellular cap domain and a C-terminal domain (CTD), collectively orchestrating ion conduction through the central pore module [19, 20]. Moreover, the central pore module and THU7-9 are connected by a long beam helix. This beam collaborates with a specialized anchor domain to transmit mechanical stimulation signals (Fig. 1b) [14, 19, 21]. Overall, these coordinated structural elements jointly enable Piezo1 to transduce mechanical forces into electrochemical signals through precise mechanogating.

## Mechanogating mechanism of Piezo1 channel

**Fig. 2:**
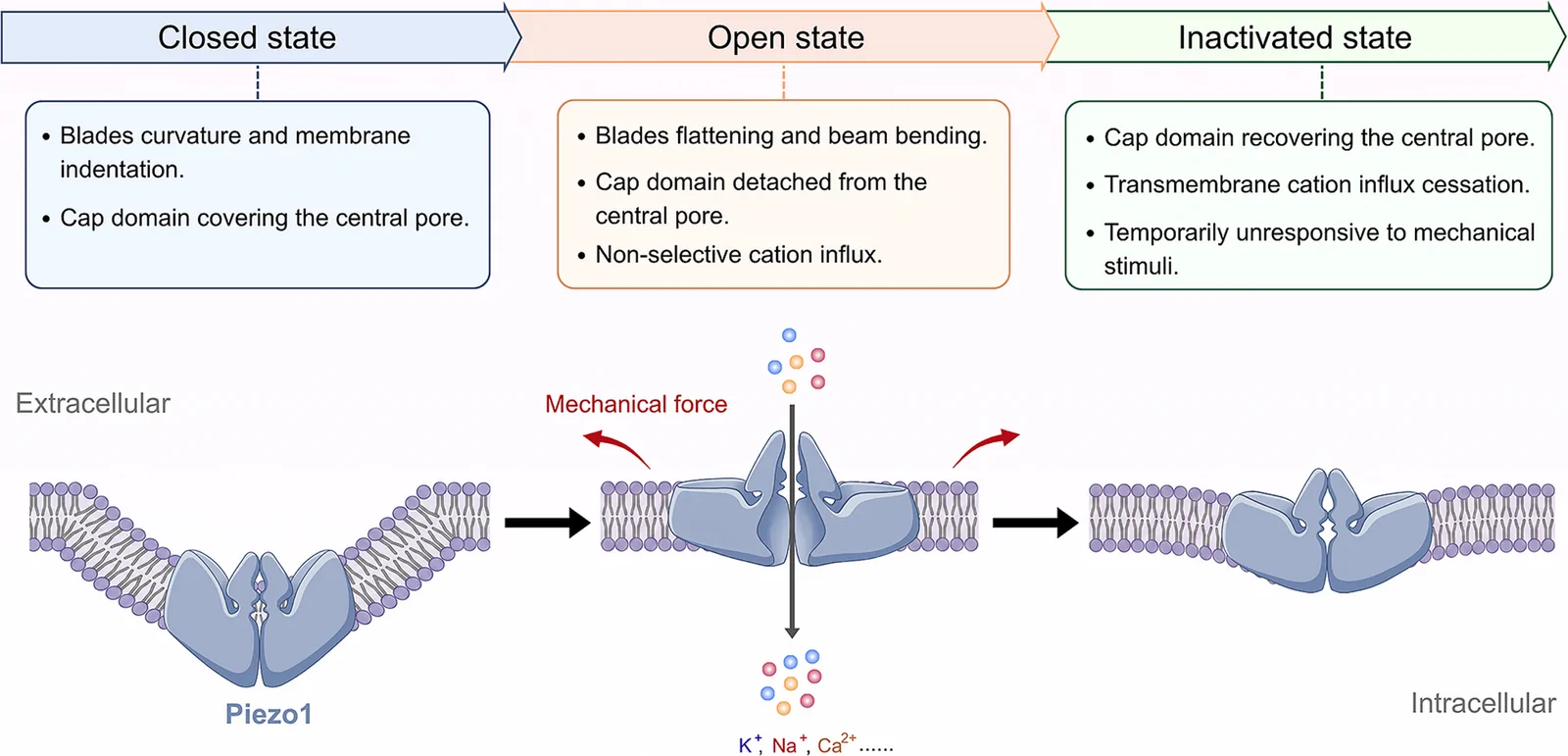
In response to mechanical stimulation, the mechanogating states of Piezo1 transition among the closed, open, and inactivated states

The mechanogating of the Piezo1 channel can be generally modeled in three distinct states: open, inactivated, and closed (Fig. 2) [22]. In the closed state, the extracellular cap of Piezo1 tightly covers the central ion conduction pore, while its trimeric blade domains are highly curved. This curvature generates a localized in‑plane indentation in the adjacent plasma membrane, forming a dome‑shaped structure [23]. The dome‑shaped curvature is stable but stores a considerable amount of elastic potential energy [24]. When the cell is subjected to stretch or fluid shear stress, the increase in membrane tension tends to flatten the dome curvature [25, 26]. Recent research has revealed that the blades of Piezo1 are induced to flatten within the membrane plane by elevated tension, expanding their in-plane area by approximately 300 nm^2^ [27]. Simultaneously, the flattening of the blades drives the bending of the intracellular beams at the pivot residues S1341, L1342, and L1345, causing the extracellular cap domain to detach and rotate [28]. Subsequently, the spring linker (cap-IH-linker) between the cap domain and the IH is compressed, leading to the opening of the hydrophobic transmembrane gate [28]. These coordinated conformational changes result in a significant flattening deformation of the overall Piezo1 structure, ultimately acting in synergy to facilitate the opening of the central ion conduction pathway, allowing for the transmembrane transport of cations such as K^+^, Na^+^, and particularly Ca^2+^. Notably, the half-maximal activation tension for the Piezo1 gating process is approximately 1.9 pN/nm, underscoring its exquisite mechanosensitivity [29]. This indicates that Piezo1 can directly perform rapid signal transduction through membrane curvature sensing without the requirement of additional auxiliary proteins [27]. However, existing experimental evidence suggests that various environmental factors and interacting proteins can modulate the activation threshold of Piezo1. For instance, the lipid composition of the membrane [30–32], the physical properties of the substrate [33, 34], the components of the extracellular matrix (ECM) [35], and environmental constraints [36, 37], have all been demonstrated to influence the sensitivity of Piezo1. Among the proteins reported to interact with Piezo1, MDFIC, MDFI, and MDFIC2 all belong to the MyoD family inhibitor proteins, are currently supported by the strongest evidence, as they represent the only Piezo1-interacting proteins whose structural basis has been resolved [38–40]. Their lipidated C-terminal helices have been demonstrated to laterally insert into the pore module of Piezo1, substantially increasing the threshold for mechanical activation and slowing channel inactivation [38]. Additionally, stomatin-like protein 3 (Stoml3) has been shown to sensitize Piezo1, while polycystin-2 (PC2) and platelet endothelial cell adhesion molecule 1 (PECAM1) may inhibit the mechanosensitivity of Piezo1 [41–43]. Furthermore, cytoskeletal proteins that regulate membrane stiffness (e.g., actin, filamin A) as well as cell adhesion-related components particularly integrins and their associated focal adhesions (FAs), have also been shown to play a crucial role in Piezo1-mediated mechanotransduction [44–46]. Specifically, integrins anchor cells to ECM and assemble into FAs, which concentrate key signaling molecules including focal adhesion kinase (FAK) [47]. Piezo1 could be recruited by mechanical forces transmitted through integrins and FAs, thereby modulating local mechanotransduction [17]. In turn, local Ca^2+^ influx mediated by activated Piezo1 could also regulate FAK phosphorylation and FAs dynamics [48]. Following activation, the linkers (including the OH–cap linker and cap-IH linker), which are in a compressed conformation, tend to spontaneously return to a high-entropy, low-free-energy extended state [49]. This restorative transition drives the repositioning of the cap domain, leading to the closure of the cap gate and transmembrane gate, thereby triggering the rapid inactivation of the Piezo1 channel. Piezo1 inactivation is also influenced by various factors. Specifically, depolarizing potentials slow down its inactivation, whereas acidic environments or low temperatures enhance its inactivation [50]. In addition, cell adhesion molecule 1 (CADM1) has been identified as a proximal protein of Piezo1, specifically slowing down the inactivation kinetics of Piezo1 and prolonging the channel open time [51]. Precisely due to the intrinsic complexity of native cellular microenvironments, Piezo1 exhibits slow inactivation in various native cells, including mouse embryonic stem cells (mESCs) [52], adipocytes [53], ECs [54], myoblasts and fibroblasts [38], distinct from the rapid inactivation observed in heterologous expression systems. In summary, the mechanogating of Piezo1 involves a series of coordinated protein conformational changes driven by membrane tension, ensuring that cells can precisely perceive and respond to alterations in their physical microenvironment.

## Physiological and cellular functions of Piezo1

**Fig. 3:**
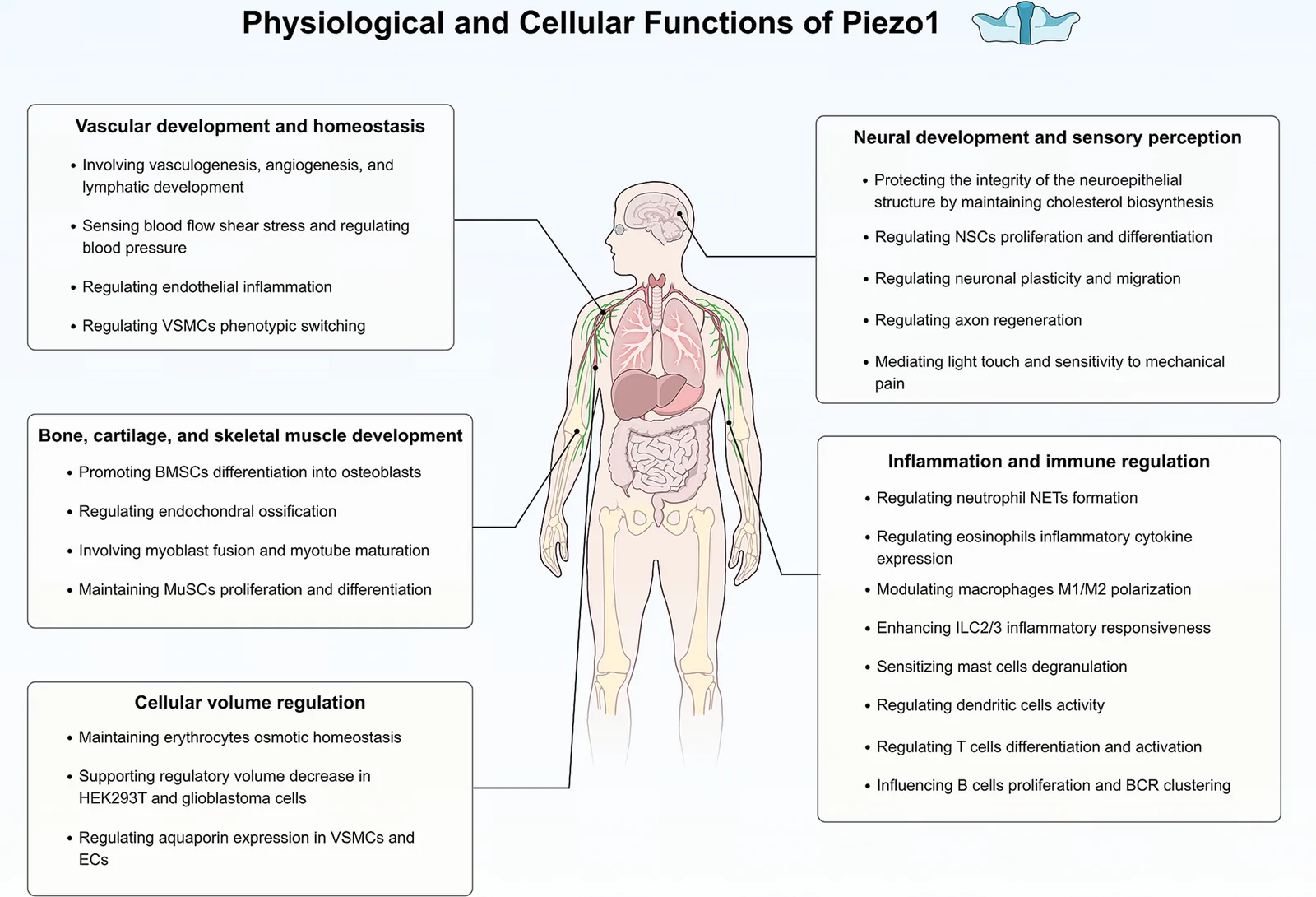
Summary of the physiological and cellular functions of Piezo1. Piezo1 is widely expressed across various tissues and cell types in mammals, where it exerts diverse functions including vascular development and homeostasis, musculoskeletal development, neural development, sensory perception, immune regulation, as well as cellular volume regulation

Through virtue of unique and sophisticated structure and mechanogating mechanism, Piezo1 responds to mechanical forces, activating a rapid influx of non-selective cations with high permeability to Ca^2+^. Thus, Piezo1 transduces mechanical signals into biochemical signals, thereby participating in a variety of physiological and cellular functions. Here, the diverse roles of Piezo1 in multiple physiological and cellular functions will be discussed (Fig. 3).

### Vascular development and homeostasis

Vascular development and homeostasis involve various processes, including vasculogenesis, angiogenesis, and the maintenance of normal structure and function in adulthood [55]. Of note, as a mechanical stress sensor, Piezo1 is expressed in various cardiovascular cells, and plays a key role in vascular development and homeostasis. Firstly, Piezo1 is essential for vascular development during embryogenesis. Piezo1 gene knockout mice exhibit severe vascular development defects, accompanied by pericardial effusion, and die during mid-gestation (between embryonic days 9.5 and 14.5) [56]. Kang et al. revealed that Piezo1 in ECs can be directly activated by physiological levels of wall shear stress (WSS), or through a sphingosine-1-phosphate (S1P)-induced mechanism mediated by Src kinase [57]. Then the opening of Piezo1 channels triggers an influx of Ca^2+^. This calcium signal promotes the translocation of membrane-type 1 matrix metalloproteinase (MT1-MMP) to the cell membrane, where it activates MMP2. Both enzymes synergistically degrade the ECM, providing the necessary space and pathways for ECs migration, invasion, sprouting, and tubulogenesis [57]. Besides, Piezo1 in lymphatic endothelial cells (LECs) can be directly activated by physiological levels of oscillatory shear stress (OSS) generated by lymphatic flow. This activation specifically upregulates the expression of genes associated with lymphatic valve development (e.g., FOXC2, GATA2, CX37, and LAMA5), thereby promoting lymphatic valve formation [58, 59].

In terms of maintaining vascular homeostasis, Piezo1 functions primarily by sensing and responding to blood flow shear stress. When blood pressure increases, the enhanced shear stress activates Piezo1 in vascular endothelial cells (VECs), triggering Ca^2^⁺ influx and subsequent adenosine triphosphate (ATP) release [60]. Release of ATP activates the Gq/G11-coupled P2Y2 receptors, promoting phosphorylation of protein kinase B (AKT) and endothelial nitric oxide synthase (eNOS), thereby enhancing production of nitric oxide (NO), which is a potent vasodilatory signaling molecule [60, 61]. Additionally, research indicates that the activation of Piezo1 by blood flow shear stress induces the release of adrenomedullin. Then adrenomedullin initiates the autocrine and paracrine activation of calcitonin receptor-like (CALCRL) receptors, which subsequently elevates cyclic adenosine monophosphate (cAMP) levels in VECs. The elevation of cAMP activates protein kinase A (PKA), leading to the specific phosphorylation of eNOS, which functions synergistically with the AKT-mediated phosphorylation of eNOS to promote the production of NO [61, 62]. Beyond the regulation of NO production, Piezo1 activation in mouse mesenteric artery ECs mediates cation influx, leading to membrane depolarization. This depolarization electrically couples to adjacent vascular smooth muscle cells (VSMCs), activating voltage-gated Ca^2+^ channels and inducing vasoconstriction [63]. In addition to blood pressure regulation, Piezo1 is indispensable for maintaining the intrinsic anti-inflammatory function of the vasculature. When Piezo1 is activated by laminar shear stress in VECs, it suppresses the tumor necrosis factor-alpha (TNF-α) induced expression of intercellular adhesion molecule-1 (ICAM-1) and vascular cell adhesion molecule-1 (VCAM-1), which are key cell adhesion molecules involved in the recruitment of leukocytes [64]. Meanwhile, Piezo1 activation induces Ca^2^⁺ influx, which activates calcium/calmodulin-dependent protein kinase II (CaMKII) signaling pathway in VECs. Then, VECs exhibit upregulation of transcription factors such as Krüppel-like factor 2 (KLF2) and KLF4, which promote the expression of anti-inflammatory factors such as eNOS and thrombomodulin (THBD) [65]. In contrast, when Piezo1 is activated by oscillatory or turbulent shear stress generated by disturbed blood flow, it promotes endothelial inflammation [66, 67]. Simultaneously, pro-atherogenic genes, including cysteine-aspartic acid protease 1 (caspase-1), interleukin-1 beta (IL-1β), and NOD-like receptor thermal protein domain associated protein 3 (NLRP3) are upregulated, driving the progression of atherosclerosis [66, 68]. Notably, Piezo1 can also activate phospholipase A2 (PLA2), leading to the opening of the non-selective cation channel transient receptor potential vanilloid 4 (TRPV4), sustained Ca^2+^ influx, and subsequent ECs injury [69]. Moreover, disturbed blood flow and vascular injury have been shown to significantly upregulate the expression of Piezo1 in human and mouse VSMCs, promoting the transition of VSMCs from a contractile to a synthetic/proliferative phenotype, ultimately inducing neointimal hyperplasia [70]. To summarize, Piezo1 exerts a fundamental influence on vasculogenesis, angiogenesis, blood pressure homeostasis, and the maintenance of normal physiological functions of vasculature.

### Bone, cartilage, and skeletal muscle development

The musculoskeletal system relies on the functional synergy of bone, cartilage, and skeletal muscle [71]. Bones provide the rigid framework, cartilages ensure low-friction cushioning at bone ends, and skeletal muscles attach to the bone to drive movement through contraction and relaxation [72]. Accumulating evidence highlights the critical role of mechanical signaling in musculoskeletal development, underscoring the importance of the mechanotransduction channel Piezo1. For bone development, Piezo1 functions predominantly in bone marrow mesenchymal stem cells (BMSCs) and osteoblasts [73]. The expression of Piezo1 in BMSCs can be significantly upregulated by mechanical stretching [74]. Conversely, conditional knockout of Piezo1 in limb bud mesenchyme leads to spontaneous fractures, shortened long bones, impaired bone mass and bone formation, and increased bone resorption in neonatal mice [75]. Mechanistically, Piezo1 mediates Ca^2+^ influx in BMSCs and activates calcineurin (Ppp3ca), leading to the dephosphorylation of nuclear factor of activated T cells 1 (NFATc1), Yes-associated protein 1 (YAP1), and β-catenin, which then form a transcriptional complex that collectively drives the expression of osteogenic differentiation associated genes [75]. Additionally, Piezo1-mediated upregulation of β-catenin promotes the expression of activating transcription factor 4 (ATF4), which is a key transcription factor for bone formation, thereby enhancing the proliferation and osteogenic differentiation capabilities of BMSCs [76]. Furthermore, recent studies have shown that Piezo1 in BMSCs ensures the differentiation of BMSCs towards osteoblasts rather than adipocytes by inhibiting the C C motif ligand 2 (CCL2)-lipocalin-2 (Lcn2) inflammatory autocrine loop, thereby supporting bone development [77]. Another critical component of skeletal development is osteoprogenitor cells [78]. In particular, the specific deficiency of Piezo1 in osteoprogenitors leads to a significant reduction in trabecular and cortical bone mass in C57BL/6J mice by day 21 post-birth, as well as a decline in the serum bone formation marker procollagen type I N-terminal propeptide (PINP) at 6 weeks [75]. Intriguingly, 8 weeks old female C57BL/6J mice with osteoblast lineage-specific Piezo1 deficiency showed no discernible abnormalities in either serum bone formation markers or bone volume [79]. Bone volume maintenance in adult mice is largely unaffected by Piezo1 deficiency in mature osteoblasts, potentially due to compensation by other mechanotransduction proteins (e.g., Piezo2) or skeletal adaptation mechanisms [75]. Presumably, Piezo1 in earlier stage osteoblasts is indispensable for bone development during the active bone growth, particularly in the early postnatal and even embryonic periods.

Beyond the role in bone development, Piezo1 is crucial for regulating physiological endochondral ossification during cartilage development. Under normal conditions, Piezo1 suppresses the expression of chondrogenic genes, ensuring that growth plate-derived chondrocytes correctly differentiate into osteoblasts capable of forming secondary spongiosa [80]. Conversely, due to impaired secondary spongiosa formation, mice with cartilage-specific Piezo1 deficiency develop severe osteoporosis and spontaneous multiple fractures shortly after birth [80, 81]. However, evidence has shown that excessive mechanical loading leads to the pathological overactivation of Piezo1, which activates calcineurin and promotes the nuclear translocation of NFATc2, thereby inducing chondrocyte apoptosis and matrix degradation [81–83]. Therefore, the precise physiological regulation of Piezo1 activity is critical to strike a balance between preventing developmental deficiencies caused by loss of function and avoiding cartilage degeneration induced by its overactivation.

In skeletal muscle development, Piezo1 is essential for myoblast fusion and subsequent myotube maturation, a pivotal stage in the formation of functional muscle tissue [84]. In 2018, Tsuchiya et al. found that Piezo1 is involved in the regulation of myotube formation [85]. Subsequently, Quiroga et al. demonstrated that Piezo1 knockdown significantly downregulates the expression of the key myocyte fusion protein myomaker and reduces the fusion index. However, excessive activation of Piezo1 can also lead to a reduction in myotube diameter and an imbalance in maturation [84]. Moreover, in muscle stem cells (MuSCs) responsible for muscle growth, repair, and regeneration, Piezo1 is highly expressed and primarily localized in the quiescent MuSCs [86]. The deletion of Piezo1 leads to premature activation of MuSCs, impairing their proliferative and differentiation abilities, thereby causing significant disruption in skeletal muscle regeneration [86]. Mechanistically, Piezo1 deficiency induces compensatory upregulation of T-type voltage-gated Ca^2+^ channels, enhancing Ca^2+^ influx, which activates classical protein kinase C (cPKC) and upregulates NADPH oxidase 4 (NOX4) expression. This results in excessive production of reactive oxygen species (ROS), accumulation of DNA damage, and ultimately activates the p53/P21 signaling pathway, leading to MuSCs senescence and cell death [86]. In summary, the precise balance of Piezo1 activity is critical for coordinating the development of bone, cartilage, and skeletal muscle.

### Neural development and sensory perception

Neural development provides the foundation for sensory perception, enabling organisms to effectively detect and process stimuli from both internal and external environments [87]. Existing studies suggest that neural development is influenced by mechanical signals from the microenvironment transduced by Piezo1, including cellular traction forces, intercellular tension, and matrix stiffness [88]. For instance, higher matrix stiffness significantly inhibited the expression of downstream transthyretin (TTR) through Piezo1 activation, thereby impeding electrophysiological maturation and synapse formation of neurons [89]. Functionally, Piezo1 maintains neuroepithelial structural integrity and regulates the proliferation and multipotent differentiation of neural stem cells (NSCs) during brain development by sustaining cholesterol biosynthesis [90]. Moreover, neurogenesis is regulated by Piezo1 in astrocytes. Astrocyte-specific Piezo1 knockout in mice resulted in a decrease in the number of NSCs, neural progenitor cells, immature and mature neurons, along with a significant reduction in hippocampal volume and brain weight [91]. In terms of mechanism, Piezo1 indirectly modulated neuronal plasticity by promoting ATP release from astrocytes, which stimulated the proliferation and neurogenic differentiation of hippocampal NSCs [91]. Furthermore, in the three-dimensional (3D) microenvironment of the brain, Piezo1 activation induced the PKC-ezrin signaling cascade, which recruits actin filaments to the neuronal posterior plasma membrane and transmits contractile forces to assist neuronal migration in the 3D confined space [92]. Besides, the activation of Piezo1 at the growth cone promoted the phosphorylation of FAK, which regulated actin dynamics and determined the regenerative capacity of dorsal root ganglion (DRG) axons [92]. Additionally, recent studies have shown that retinal ganglion cells (RGCs) and brain neuroepithelial cells mediate axonal autonomous mechanosensing as well as regulate brain tissue mechanical stiffness alongside long-range chemical signaling, respectively [93]. Together, these two determinants collaboratively influence the guidance capacity of RGC axons.

Regarding sensory perception, Piezo1 exhibits low expression levels in sensory neurons and primarily serves a modulatory role [94, 95]. Specifically, it mainly influences sensitivity to light touch and mechanical pain, distinct from Piezo2, which primarily mediates tactile sensation and proprioception [96]. Recent studies have established that Piezo1 is indispensable for dynamic light touch sensation in mice, whereas it has no impact on sensitivity to punctate mechanical stimuli or temperature stimuli [97]. In the anterior cingulate cortex, a pivotal brain region for pain perception, upregulation of Piezo1 expression supports the transmission of pain signals, leading to hypersensitivity to pain [98]. Furthermore, Piezo1 may be selectively highly expressed in smaller diameter DRG neurons, where it mediates the transmission of mechanical pain signals [98, 99]. In 2022, Hill et al. demonstrated that Piezo1 is specifically highly expressed in mechanical itch-dedicated sensory neurons within the DRG of both mice and humans, where it mediates the transduction of mechanical itch signals [100]. Strikingly, widespread overexpression of Piezo1 in DRG neurons significantly reduced mouse sensitivity to mechanically induced pain. It is hypothesized that the enhanced tactile input from Piezo1 indirectly inhibits nociceptive signal transmission through the spinal gate control mechanism [101]. Interestingly, Piezo1 expressed in non-neuronal cells, such as keratinocytes and odontoblasts, can also indirectly influence sensory perception by regulating ATP release and thereby modulating the activity of adjacent sensory nerve endings, particularly under pathological conditions [102, 103]. Taken together, Piezo1 orchestrates regulatory roles in neural development, light touch, and mechanical pain perception.

### Inflammation and immune regulation

Inflammation serves as a protective defense mechanism against external stimuli, whereas immune regulation is necessary to prevent excessive inflammation from causing harm to the body [104, 105]. Previous studies have demonstrated that Piezo1 is widely expressed across various immune cell types and plays a significant role in inflammation and immune regulation [4]. On the one hand, in innate immunity, neutrophils are the primary effector cells. In neutrophils, blood flow shear stress can activate Piezo1, which in turn activates calpain (a calcium-dependent cysteine protease), promoting the formation of neutrophil extracellular traps (NETs), thereby contributing to local sterile inflammation and promoting thrombosis [106]. In addition to neutrophils, Piezo1 in eosinophils can regulate the expression of inflammatory cytokines, thereby enhancing TRPV1- and purinergic receptor–mediated calcium responses in mouse DRG [107]. Beyond granulocytes, Piezo1 also regulates the functions of macrophages, which are capable of pathogen phagocytosis and antigen presentation [108]. In macrophages, increased ECM stiffness enhances Piezo1 activation and promotes polarization toward the M1 phenotype, which is a pro-inflammatory profile [109]. Enhanced M1 polarization of macrophages could strengthen anti-infective and antitumor immunity, but may also lead to excessive inflammation and self-damage [110]. Furthermore, innate lymphoid cells (ILCs) represent a class of innate immune cells that lack specific antigen receptors and are primarily categorized into three functional groups [111]. Specifically, ILC1 mainly secretes IFN-γ against intracellular pathogens, ILC2 produces IL-5 and IL-13 to mediate immunity against parasites and allergic inflammation, and ILC3 releases IL-17 and IL-22 in response to extracellular bacteria and fungi [111, 112]. In ILC3, Piezo1 activation promotes its proliferation and the secretion of IL-17A. While in ILC2, Piezo1 can positively regulate IL-13 synthesis at the translational level, thereby promoting type 2 immune responses [113, 114]. Notably, among various innate immune cells, mast cells (MCs) are intrinsically sensitive to mechanical stimuli and serve as a bridge between innate and adaptive immunity [115]. Piezo1 expression in MCs can be upregulated by IL-33, enhancing their sensitivity to mechanical stimuli. This sensitization may promote MCs degranulation, in an allergic environment, thereby exacerbating inflammatory responses [116]. Besides, dendritic cells (DCs), another immune cell bridging innate and adaptive immunity, serve as professional antigen-presenting cells (APCs) [117]. Evidence suggested that bone marrow-derived dendritic cells (BMDCs) cultured under conditions of lower substrate stiffness exhibit significantly reduced levels of pro-inflammatory cytokines. In contrast, the activation of Piezo1 can enhance the activity of BMDCs [118]. Moreover, mechanical stiffness or inflammatory signals can activate Piezo1 in DCs, leading to increased IL-12 and decreased transforming growth factor-β (TGF-β) production, which indirectly promotes the differentiation of naïve CD4^+^ T cells into T helper 1 (Th1) while inhibiting their differentiation into regulatory T (Treg) cells [119].

On the other hand, in adaptive immunity, besides indirectly regulating T cell differentiation through DCs, Piezo1 can directly modulate T cell activation. T cell activation primarily depends on the engagement of the T cell receptor (TCR) with major histocompatibility complex (MHC) molecules expressed on APCs [120]. TCR signaling can be optimized by Piezo1-driven Ca^2+^ influx through the activation of calpain and the cortical actin cytoskeleton, thereby promoting T cell activation [121]. Additionally, T cell activation can be enhanced by fluid shear stress mediated by Piezo1 [122]. Interestingly, Jairaman et al. found that Piezo1 had a selective inhibitory effect on Treg cells without affecting T lymphocyte activation or effector T cell function. Mechanistically, Piezo1 inhibits TGF‑β, a key cytokine for the differentiation and survival of Treg cells, thereby limiting their expansion [123–125]. Aside from T cells, B cells are the primary adaptive immune cells responsible for mediating humoral immune responses. Functionally, B cell activation is predominantly initiated by the binding of antigens to the B cell receptor (BCR), which is influenced by Piezo1 [126]. Piezo1-deficient B cells exhibit a markedly reduced proliferative capacity in response to membrane-presented antigen stimulation, accompanied by significant decreases in BCR clustering and F-actin polymerization on anti-Ig-attached planar lipid bilayers (PLBs) [127]. Overall, Piezo1 transduces mechanical signals in both innate and adaptive immunity, regulating the inflammatory response and maintaining immune homeostasis.

### Cellular volume regulation

Cell volume regulation is a biological process essential for maintaining cellular homeostasis and function [128]. The concept of Piezo1 as a mechanosensitive channel involved in cell volume regulation originated from studies on erythrocytes [129]. Specifically, when erythrocytes experience mechanical force, Piezo1 channels are activated, mediating the influx of Ca^2+^ [130]. The increase in intracellular Ca^2+^ directly activates the Gardos channel (KCa3.1), which facilitates the rapid efflux of K^+^ out of the cell along the concentration gradient. The efflux of K^+^ causes a significant reduction in intracellular osmotic pressure, which ultimately leads to erythrocyte dehydration and a subsequent decrease in cell volume [131]. Therefore, Piezo1 deficiency in erythrocytes leads to overhydration, increased cell volume, and significantly elevated osmotic fragility, thereby compromising cell survival [132]. In addition, an in vitro study found that the capacity for regulatory volume decrease (RVD) in HEK293T cells following osmotic shock-induced swelling is highly correlated with Piezo1 expression levels [133]. Mechanistically, Piezo1-mediated Ca^2+^ influx may positively regulate the activity of volume-regulated anion channels (VRAC), promoting Cl⁻ efflux, and facilitates RVD in concert with K^+^ efflux [133, 134]. Similarly, in human glioblastoma (GBM) cells, Piezo1 is involved in the activation of large-conductance and intermediate-conductance Ca^2+^-activated K^+^ (BKCa and IKCa) channels, which support the process of RVD [135, 136]. Furthermore, in cell types such as VSMCs and ECs, Piezo1 has been demonstrated to modulate the membrane expression of aquaporins, thereby regulating cell volume [137, 138]. To sum up, Piezo1 couples Ca^2^⁺ influx with downstream channel proteins in various cell types, dynamically regulating cell volume.

## Piezo1 in human diseases and pathologies

**Fig. 4:**
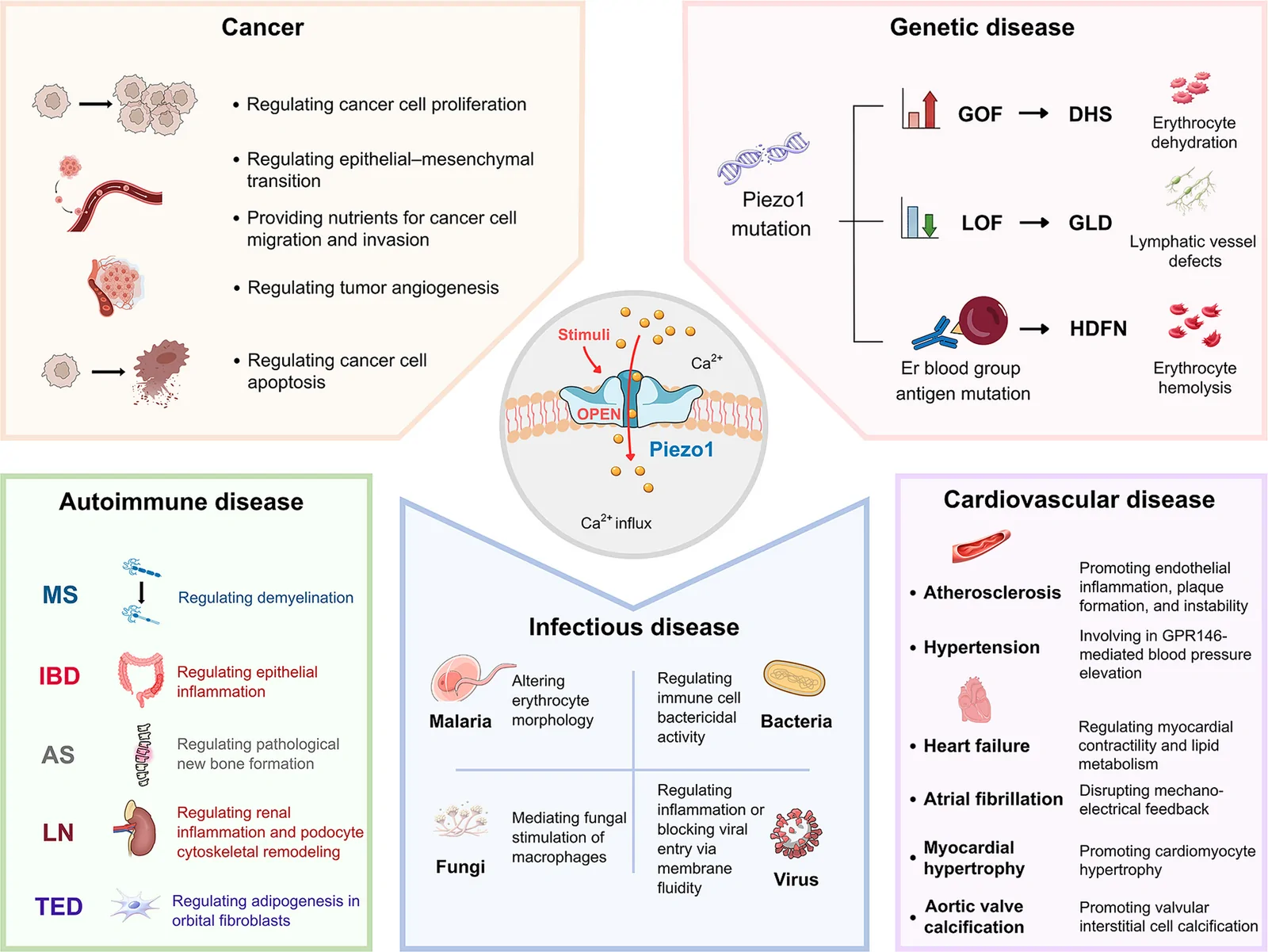
Mutations or dysregulation of Piezo1 drive the onset and progression of various diseases. Certain Piezo1 mutations are responsible for the development of genetic disorders, while its dysregulation contributes to the pathogenesis of genetic disorders, cardiovascular diseases, infectious diseases, autoimmune diseases, and cancer

Given the critical role of Piezo1 in physiological and cellular functions, it has emerged as an important regulator in a variety of human diseases. Increasing evidence indicates that Piezo1 aberrant expression or dysfunction is closely associated with the occurrence and progression of genetic disorders, cardiovascular disease, infectious diseases, autoimmune diseases, and cancer (Fig. 4). In this section, the specific roles of Piezo1 in the progression and pathologies of these diseases will be summarized.

### Piezo1 mutation-associated genetic disease

#### Dehydrated hereditary stomatocytosis

Dehydrated hereditary stomatocytosis (DHS), also known as hereditary xerocytosis (HX), is a rare autosomal dominant inherited disorder characterized by increased cation permeability of the erythrocyte membrane, leading to cellular dehydration and hemolytic anemia [139]. In 2012, Zarychanski et al. first identified heterozygous missense mutations in Piezo1 (encoded by *FAM38A*) at residues 2225 (M2225R) or 2456 (R2456H) through copy number analyses, linkage analysis, whole-exome sequencing, followed by Sanger sequencing in DHS families [140]. In 2013, Andolfo et al. further expanded the mutation spectrum and performed functional characterization of the R2456H mutation as well as a novel variant at position 2488 (R2488Q). They found that these mutations increase Piezo1 channel activity, leading to enhanced efflux of potassium accompanied by water loss in erythrocytes, thereby resulting in cellular dehydration and hemolytic anemia [141]. Moreover, accumulating evidence indicates that the role of Piezo1 in DHS is not limited to mature erythrocytes. For example, aberrant activation of Piezo1 can delay erythroid differentiation, regulate iron metabolism in hepatocytes and macrophages leading to non–transfusion-dependent iron overload, or contribute to perinatal edema by affecting lymphatic valve development and maintenance [142, 143]. Collectively, gain-of-function (GOF) mutations in Piezo1 drive the diverse clinical manifestations of DHS by disrupting erythrocyte hydration homeostasis, as well as processes related to hematopoiesis, iron metabolism, and lymphatic development.

#### Generalized lymphatic dysplasia

Generalized lymphatic dysplasia (GLD) is a rare congenital anomaly of the lymphatic system, characterized by widespread systemic lymphedema, chylous effusions, and profound structural and functional defects of the lymphatic vasculature [144]. In 2015, Fotiou et al. performed whole-exome sequencing in six families with GLD presenting with nonimmune fetal hydrops (NIHF), and identified a diverse array of Piezo1 mutations, including nonsense mutations (e.g., E1630X, E755X, Q2228X), missense mutations (e.g., V2171F, L939M), and splice-site mutations (e.g., c.3796 + 1G > A, c.1669 + 1G > A) [145]. These mutations are biallelic loss-of-function (LOF) variants (homozygous or compound heterozygous) with autosomal recessive inheritance, leading to markedly reduced expression or impaired function of the Piezo1 protein and resulting in lymphatic developmental defects in affected individuals [145]. In the same year, Lukacs et al. reported compound heterozygous mutations in Piezo1 in patients with GLD, including a splice-site mutation (c.3455 + 1G > A) and a missense mutation (c.6085G > C), which resulted in reduced Piezo1 expression and a marked decrease in current density induced by both mechanical and chemical activation [146]. In 2022, Han et al. found that a homozygous missense mutation in the Piezo1 gene (c.5162C > G) markedly reduces its expression, leading to autosomal recessive lymphatic malformation type 6 (LMPHM6), a subtype of GLD [147]. Notably, GLD is an important cause of NIHF, and a recent study has shown that Piezo1 mutations represent the most common monogenic cause of NIHF identified by prenatal exome sequencing [148]. Taken together, LOF mutations in Piezo1 impair its expression or mechanotransduction capacity, disrupt lymphatic system development, and constitute a critical genetic basis for the development of GLD.

#### Hemolytic disease of the fetus and newborn

Hemolytic disease of the fetus and newborn (HDFN) is a condition caused by maternal–fetal blood group incompatibility, in which maternal antibodies cross the placenta and target fetal or neonatal erythrocytes, leading to hemolysis, anemia, and jaundice [149]. In 2023, Crew et al. utilized whole-exome sequencing, clustered regularly interspaced short palindromic repeats (CRISPR)-mediated gene editing, and immunoprecipitation assays to identify Piezo1 as the carrier molecule for Er blood group antigens [150]. Their findings characterized several pivotal mutations, including those determining Era/Erb antigen polymorphism (p.Gly2394Ser), Er3^−^ (p.Glu2392Lys), Er4^−^ (p.Glu2407Gln), and Er5^−^ (p.Arg2245Gln). Among these variants, the Er4^−^ and Er5^−^ phenotypes exhibit clear pathogenicity. They can trigger the production of anti-Er4 and anti-Er5 antibodies, respectively, leading to HDFN and resulting in adverse pregnancy outcomes [150]. Additionally, in 2025, Jiang et al. identified a hydrops fetalis-associated missense mutation in Piezo1 (p.Leu322Pro) [151]. This mutation is located within the THU2 domain of Piezo1 and selectively impairs mechanical activation without compromising membrane localization or pharmacological activation [151].

### Piezo1 and cardiovascular disease

#### Atherosclerosis

Atherosclerosis is a highly prevalent chronic vascular disorder and represents the leading cause of cardiovascular morbidity and mortality worldwide. As previously described regarding Piezo1 in vascular homeostasis, turbulent shear stress activates Piezo1, triggering endothelial inflammation and upregulating pro-atherogenic genes [66, 67]. Moreover, in the aortas of atherosclerotic model mice, Piezo1 expression is markedly upregulated, particularly in endothelial regions exposed to disturbed flow [67]. Conversely, in VECs exposed to turbulent shear stress in endothelial-specific Piezo1 knockout mice, VCAM-1 expression and plaque formation during atherosclerosis are significantly reduced [152]. Mechanistically, turbulent shear stress induces Piezo1 activation in vascular endothelial cells via Gq/G11 (heterotrimeric G protein), promoting integrin α5 activation and triggering downstream pro-inflammatory signaling pathways [152]. Alternatively, it can induce FAK/Src phosphorylation through the Ca^2+^/CaM/CaMKII signaling pathway, subsequently activating YAP and ultimately promoting endothelial inflammation [67]. In addition, Konishi et al. reported that Piezo1 expression in myofibroblasts within the fibrous caps of plaques was significantly elevated in human patients with carotid atherosclerosis [153]. Simultaneously, Piezo1 upregulation is closely associated with indicators of plaque instability, including plaque rupture, thin-cap fibroatheroma, and microcalcifications within the fibrous cap [153]. Altogether, aberrant upregulation of Piezo1 promotes atherosclerosis and compromises plaque stability.

#### Heart failure

Heart failure refers to the impaired pumping function of the heart, resulting from various cardiovascular pathological conditions [154]. Current studies have indicated that aberrant Piezo1 is associated with the progression of cardiomyopathies related to heart failure. In 2021, Jiang et al. found that Piezo1 expression was significantly increased in the myocardial tissue of patients with hypertrophic cardiomyopathy and in mouse models of heart failure [155]. Functionally, Piezo1 responds to the stretching of cardiomyocytes, inducing rapid Ca^2^⁺ influx and promoting ROS production, thereby enhancing myocardial contractility [155]. Therefore, the upregulation of Piezo1 in the myocardial tissue of heart failure patients, may represent a compensatory response aimed at mitigating cardiac dysfunction. Furthermore, in 2025, Fan et al. demonstrated that reducing the activation threshold of Piezo1 and prolonging its open state impaired lipid metabolism in cardiomyocytes. This impairment inhibited fatty acid β-oxidation, induces myocardial lipotoxicity, and may ultimately contribute to the development of heart failure [156]. Overall, dysregulation of Piezo1 may serve as a relevant indicator of heart failure.

#### Other cardiovascular disease

In other cardiovascular diseases, including atrial fibrillation [157], myocardial hypertrophy [158], aortic valve calcification [159], and hypertension [160], Piezo1 has also been demonstrated to serve as a mechanochemical transduction hub deeply involved in their development and progression. For instance, in patients with atrial fibrillation, both the expression and activity of Piezo1 are significantly upregulated in atrial fibroblasts, while the activity of BKCa channels, which is coupled with Piezo1 function, is decreased. This may lead to heightened atrial mechanosensitivity and disordered mechano-electrical feedback, thereby increasing AF susceptibility and maintenance, or even promoting atrial fibrosis [157]. Furthermore, in cardiac tissue, Piezo1 is upregulated and enriched near T-tubules and intercalated discs under pressure overload [161]. Upon activation, Piezo1-mediated Ca^2+^ influx promotes the transcription of hypertrophy-related factors, inducing cardiomyocyte hypertrophy [158]. Similarly, in calcified aortic valves, Piezo1 expression is elevated. It can be activated by OSS, subsequently upregulating glutaminase 1 (GLS1)-mediated glutaminolysis. The acetyl-CoA generated from glutamine metabolism enhances the expression of osteogenic transcription factors, ultimately driving valvular interstitial cell calcification [159]. Additionally, Piezo1 in VSMCs is positively regulated by G protein-coupled receptor 146 (GPR146), which is a novel pressure receptor in VSMCs induced by high hydrostatic pressure. Piezo1 deficiency in VSMCs can significantly reverse GPR146-mediated hypertension in model mice [160]. Taken together, the mechanotransduction function of Piezo1 involves as a critical driver in the initiation and progression of various cardiovascular diseases.

### Piezo1 and infectious disease

#### Parasitic infection (Malaria)

Malaria is a parasitic infection disease caused by Plasmodium parasites and transmitted through the bites of infected mosquitoes [162]. Plasmodium parasites invade human hepatic cells and erythrocytes leading to liver damage and anemia, even multi-organ failure and death in severe cases [163, 164]. In 2018, Ma et al. discovered that the frequency of the GOF mutation E756del of Piezo1 in the African population was as high as 18%. The E756del mutation causes erythrocyte dehydration and reduced cellular volume, but significantly inhibits the invasion and proliferation of *Plasmodium falciparum* in vitro [165]. Subsequently in 2020, Nguetse et al. demonstrated that heterozygous E756del is associated with a reduced incidence of severe malaria in human populations [166]. Mechanistically, the expression of Plasmodium falciparum erythrocyte membrane protein 1 (PfEMP-1) was significantly reduced on the surface of infected Piezo1 E756del heterozygous erythrocytes. This facilitated their recognition and clearance by the spleen, thereby reducing the pathogenicity of *Plasmodium falciparum* [166]. Furthermore, in 2023, Lohia et al. reported that Piezo1 activation leads to erythrocyte dehydration, resulting in the deformation of its biconcave structure. This structural change potentially reduces the effective surface area required for the attachment and internalization of the *Plasmodium falciparum*, thereby preventing its efficient invasion [167]. Collectively, enhancing Piezo1 activity in erythrocytes may serve as a viable strategy for malaria resistance.

#### Bacterial infection

Bacterial infection is a pathological condition resulting from the invasion and proliferation of pathogenic bacteria within a host, or the subsequent release of toxins [168]. Recent research has revealed that the Gram-negative bacterial ligand lipopolysaccharide (LPS) triggers the assembly of Toll-like receptor 4 (TLR4) and Piezo1 into a functional complex in macrophages. In this complex, Piezo1 mediates influx to activate downstream signaling pathway, thereby promoting phagocytosis and mitochondria-phagosome juxtaposition and ROS production to bolster bacterial clearance [169]. Nevertheless, in epithelial monolayer cells, LPS recruits Piezo1 to assemble with TLR2/4, regulating phospho-myosin light chain 2 (p-MLC) to remodel the cytoskeleton and accelerate bacterial endocytic invasion [170]. Moreover, during *Salmonella* infection, LPS released by *Salmonella* activates TLR4, which locally activates PIEZO1 to regulate host cell membrane remodeling processes and facilitate *Salmonella* invasion [171]. Among various bacterial infectious diseases, sepsis is one of the most critical and lethal conditions [172]. During the progression of sepsis, the expression of Piezo1 in intestinal tissues significantly increases, triggering mitochondrial calcium overload, which leads to intestinal barrier dysfunction and exacerbates the translocation of bacteria or toxins into the blood circulation, exacerbating the pathological process [173, 174]. Intriguingly, evidence also suggested that Piezo1 activation in the mesenteric microvascular endothelium could rescue the suppressed acetylcholine-induced vasodilation typically observed in sepsis, thereby protecting the intestine [175]. Furthermore, during lung injury caused by *Pseudomonas aeruginosa* infection, polymorphonuclear leukocytes (PMNs) migrate to the infection site in the lungs through endothelial adherens junctions (AJs) [176]. When passing through the narrow AJs, the high membrane tension can activate Piezo1. Subsequently, Piezo1 promotes the stabilization of hypoxia-inducible factor 1-α (HIF-1α), which upregulates NOX4 expression, promoting ROS production to enhance the bactericidal capacity in PMNs [176]. To summarize, the mechanotransduction role of Piezo1 in bacterial infections may have contrasting effects, depending on the specific cell types involved.

#### Fungal infection

Compared to bacterial infections, fungal infections typically develop more slowly and are more prone to resistance issues [177]. *Candida albicans* is a common opportunistic fungal that normally colonizes mucosal surfaces but can cause local or systemic infections in immunocompromised individuals [178]. During infection, *Candida albicans* transition from a commensal yeast form to a more rigid and invasive hyphal form [179]. In this transition, Piezo1 in innate immune cells can be activated by mechanical forces. On one hand, it upregulates the expression of C-type lectin receptors (CLRs). While on the other hand, it activates nuclear Dbf2-related kinase 1/2 (NDR1/2), promoting apoptosis-associated speck-like protein containing a CARD (ASC) protein phosphorylation and facilitating NLRP3 inflammasome assembly and activation. Ultimately, both mediate the release of pro-inflammatory cytokines and the recruitment of immune cells, thereby enhancing fungal clearance [180]. In addition, Piezo1 expression is significantly upregulated in the corneas of both fungal keratitis patients and mouse models [181]. At the molecular level, fungal spore stimulation induces Piezo1 upregulation, which activates RhoA-GTP and upregulates Ras-related C3 botulinum toxin substrate 1/2 (Rac1/2), thereby enhancing the phagocytic capacity of macrophages toward the spores. Simultaneously, Piezo1 regulates the assembly of the Pyrin inflammasome, promoting caspase-1 activation and the release of pro-inflammatory cytokines [181]. In brief, current research suggested that Piezo1 supports antifungal immunity.

#### Viral infection

Viral infections consistently represent a major threat to human health, leading to public health emergencies and global pandemics [182]. Following the outbreak of Coronavirus Disease 2019 (COVID-19) pandemic caused by severe acute respiratory syndrome coronavirus 2 (SARS-CoV-2), the role of Piezo1 in viral infections has attracted increasing attention. In 2023, Yang et al. found that the spike protein receptor-binding domain (S-RBD) of SARS-CoV-2 could bind to angiotensin-converting enzyme 2 (ACE2), leading to the upregulation of Piezo1. This upregulation resulted in an abnormal elevation of intracellular Ca^2+^ levels, which subsequently promoted apoptosis in pulmonary VECs and exacerbated vascular injury [183]. Accordingly, inhibiting the activation of Piezo1 helped alleviate pulmonary vascular endothelial injury during SARS-CoV-2 infection. In 2025, Wang et al. revealed that during influenza A virus (PR8, H1N1) infection, Piezo1 directly regulated the formation of NETs by sensing fluctuations in magnesium ion concentrations, thereby promoting M1 macrophage polarization [184]. Based on this, inhibiting Piezo1 may alleviate tissue damage caused by inflammatory infiltration, but it could also weaken the antiviral clearance ability. Remarkably, a recent study demonstrated that mechanical forces could mediate broad-spectrum antiviral innate immune defense by activating Piezo1, independent of the canonical interferon pathway [185]. Specifically, the cation influx mediated by Piezo1 significantly reduces the membrane fluidity of host cells, restricting the lateral diffusion of membrane proteins and lipids, thereby physically impeding the viral invasion into host cells [185]. Consequently, the physical barrier function of the cell membrane regulated by Piezo1 may serve as a novel strategy for combating viral infections.

### Piezo1 and autoimmune disease

#### Multiple sclerosis

Multiple sclerosis (MS) is a common chronic inflammatory demyelinating disease of the central nervous system (CNS) and is classified as an autoimmune disease [186]. Existing research on the role of Piezo1 in MS has predominantly focused on its involvement in demyelination and the regulation of oligodendrocyte function, both of which are central to MS pathology [187]. For example, Velasco-Estevez et al. found that Piezo1 is highly expressed in myelinated neurons, and its aberrant activation triggers demyelination in ex vivo mouse cerebellar slice cultures. Conversely, inhibition of Piezo1 significantly attenuated psychosine-induced demyelination and axonal damage [188]. A subsequent investigation revealed that Piezo1 was downregulated in the white matter of MS patient brains. Notably, Piezo1 mRNA levels did not differ significantly between MS lesions and normal-appearing white matter, suggesting that the Piezo1 downregulation occurred at the whole-brain level rather than being confined to demyelinated or degenerative regions. Simultaneously, the activation of Piezo1 in MO3.13 oligodendrocytes (a human oligodendroglial cell line) inhibited their proliferation and migration [189]. Since the proliferation and migration of oligodendrocytes are critical for remyelination, the downregulation of Piezo1 may facilitate the migration of oligodendrocytes to demyelinated regions, thereby promoting remyelination [190]. Moreover, recent studies have revealed a regulatory role for Piezo1 in T cells during the pathogenesis of MS. In the experimental autoimmune encephalomyelitis (EAE) model, T cell–specific deletion of Piezo1 markedly attenuated disease severity. This protective effect was attributed to enhanced TGF-β signaling and an expansion of the Treg compartment [189]. Based on these findings, the downregulation of Piezo1 expression in MS brains may serve as a feedback mechanism, allowing cells to adapt to a progressively stiffening microenvironment by preserving their mechanosensory properties and enhancing oligodendrocyte proliferation and migration. In a nutshell, Piezo1 has the potential to be an effective target to alleviate CNS demyelination in the development of MS.

#### Inflammatory bowel disease

Inflammatory bowel disease (IBD) comprises a group of chronic, relapsing autoimmune diseases, with Crohn’s disease (CD) and ulcerative colitis (UC) as the principal subtypes [191, 192]. Existing research revealed that the expression levels of Piezo1 are significantly higher in both active CD and UC patients compared to healthy individuals, a finding also corroborated by the dextran sulfate sodium (DSS)-induced murine colitis model [193, 194]. In contrast, DSS-induced colitis was alleviated by Piezo1 inhibition, as evidenced by reduced weight loss, increased colon length, and decreased disease activity index (DAI) scores. In particular, inhibiting Piezo1 downregulated the expression of pro-inflammatory mediators such as IL-1β, IL-6, and prostaglandin-endoperoxide synthase 2 (PTGS2) in colon tissues. Moreover, activation of Piezo1 was shown to promote the proliferation of mouse ILCs, and to enhance their expression of IL-17A. Then the activation of ILC3s exacerbates inflammatory cell infiltration and epithelial damage in colitis, which can be suppressed by inhibiting Piezo1 [194]. Similarly, in HT29 cells (a human colorectal cancer cell line), knockdown of Piezo1 led to reduced levels of inflammatory cytokines, including IL-6 and TNF-α [193]. Remarkably, Wang et al. found that mice with myeloid-specific deletion of Piezo1(Piezo1^ΔLysM^) significantly alleviated the severity of DSS-induced chronic colitis, accompanied by marked reductions in macrophage infiltration (CD68^+^ and F4/80^+^) and M1 polarization (CD86^+^), while M2 polarization (CD163^+^) remained unchanged. Interestingly, macrophage-specific deletion of Piezo1 had no significant effect on DSS-induced acute colitis, and heterozygous Piezo1 knockout mice did not exhibit notable improvement in chronic colitis [195]. In conclusion, the expression of Piezo1 is generally upregulated in patients with IBD, particularly in ILC3s and macrophages, where its elevated activation is closely associated with the progression of chronic IBD.

#### Ankylosing spondylitis

Ankylosing spondylitis (AS) is an autoimmune disease characterized by chronic inflammation of the spine and sacroiliac joints [196]. Through bulk RNA sequencing and immunofluorescence staining, Chen et al. found that Piezo1 is mainly expressed in osteo-chondral lineage cells (OLCs) and upregulated in the ligaments and entheses of human AS patients [197]. The upregulation was further validated in a murine model of collagen antibody-induced arthritis (CAIA), where mice were intraperitoneally injected with a mixture of Arthrogen-CIA monoclonal antibody and LPS. In the CAIA model, although pharmacological inhibition of Piezo1 did not significantly alter inflammatory scores, it significantly inhibited entheseal new bone formation in the mice’s hind paws. Similarly, in the CAIA model with ablation of Piezo1 in Col2a1^+^ chondrocytes (Col II ^CreERT^; Piezo1^−/−^), Piezo1 deficiency did not ameliorate inflammation but did attenuate the ankylosing phenotype [197]. Furthermore, chondrogenesis and osteogenesis are essential for the progression of ankylosis in AS, and both processes have been demonstrated to be regulated by Piezo1 during entheseal new bone formation [197, 198]. Altogether, Piezo1-mediated mechanotransduction facilitates entheseal pathological bone formation in AS.

#### Lupus nephritis

Lupus nephritis (LN) is renal damage caused by systemic lupus erythematosus (SLE), which is one of the most common and severe visceral complications of SLE [199]. LN is primarily caused by the deposition of immune complexes in the kidneys, triggering an inflammatory response that leads to damage of the glomeruli, renal tubules, interstitium, and renal vasculature [200]. Recently, Rong et al. found that Piezo1 is significantly upregulated in the podocytes of both human LN patients and LN mice. In lupus-prone MRL/lpr mice, pharmacological activation of Piezo1 resulted in severe proliferative glomerulonephritis, perivascular cell infiltration, and tubulointerstitial damage, accompanied by podocyte injury and elevated serum creatinine and blood urea nitrogen (BUN) levels. Concurrently, increased infiltration of inflammatory cells and elevated levels of inflammatory cytokines such as IL-1β, IL-6, and TNF-α were observed in mice kidneys [201]. Furthermore, in the pristane-induced LN model, podocyte-specific Piezo1 knockout mice exhibited milder glomerular lesions, reduced proteinuria, and less pronounced podocyte foot process fusion (a key marker of podocyte injury). However, no significant changes were observed in serum creatinine, BUN levels, or pro-inflammatory cytokines, indicating that the deficiency of Piezo1 specifically in podocytes is insufficient to improve overall kidney function or immune infiltration [201]. Furthermore, activation of Piezo1 significantly induces cytoskeletal remodeling in podocytes, characterized by a reduction in stress fibers and the formation of actin-rich lamellipodia following Piezo1 activation [201, 202]. This cytoskeletal remodeling may compromise the integrity and stability of the glomerular filtration barrier, thereby exacerbating the pathological process of LN. Collectively, the role of Piezo1 in the pathology of LN may be somewhat specific, primarily focusing on podocyte-related injury and dysfunction.

#### Thyroid eye disease

Thyroid eye disease (TED), or thyroid-associated ophthalmopathy (TAO), is an organ-specific autoimmune disease primarily secondary to hyperthyroid Graves’ disease (GD) [203]. Generally, the pathognomonic features of TED encompass orbital inflammation, proliferation of orbital fibroblasts (OFs), differentiation of adipocytes, excessive production of hyaluronic acid, and eventual tissue fibrosis [204]. These pathological changes induce elevated intraorbital pressure and altered mechanical properties of orbital tissues, suggesting potential activation and functional involvement of the mechanosensitive channel Piezo1 in TED progression. Recently, Galgoczi et al. revealed comparable Piezo1 expression levels in orbital connective tissues and OFs between TED patients and healthy individuals [205]. Afterward, in the OFs cell culture experiments, pharmacological activation of Piezo1 inhibited adipogenesis in TED OFs, as evidenced by the downregulation of early adipogenic regulators CCAAT/enhancer-binding protein β (C/EBPβ) and C/EBPδ, as well as late adipogenic regulators peroxisome proliferator-activated receptor γ (PPARγ) and C/EBPα [205, 206]. Interestingly, intervention with Piezo1 activation during the lipid induction phase (the first 4 days of a 12-day period) had little effect on lipid accumulation in TED OFs, suggesting that the timing of Piezo1 activation modulates its inhibitory effect on adipogenesis [205]. Nevertheless, since this study primarily based on an in vitro OFs model, it cannot replicate the complex in vivo environment, such as increased intraorbital pressure and changes in tissue mechanical stiffness. In short, activation of Piezo1 has the potential to mitigate the progression of TED by inhibiting adipogenesis.

### Piezo1 and cancer

Cancer refers to a diverse group of diseases characterized by uncontrolled cellular proliferation and the capacity for tissue invasion and metastasis, which ultimately disrupt normal physiological functions and pose a significant threat to life [207]. In general, Piezo1 is upregulated in most solid tumors, including gastric cancer [208–210], colorectal cancer [211–213], oral cancer [214], hepatocellular carcinoma [215–218], esophageal cancer [219], bladder cancer [220, 221], prostate cancer [222, 223], breast cancer [224–226], ovarian cancer [227, 228], cervical cancer [229–231], and GBM [232–234], where it promotes multiple malignant phenotypes and drives cancer progression (Table 1). Firstly, Piezo1-mediated Ca^2+^ influx can activate cell proliferation–related signaling pathways, promoting cancer cell proliferation. For example, in gastric cancer cells, high Piezo1 expression promotes the expression of pro-proliferative cyclins, thereby accelerating cancer cell proliferation [209]. Secondly, Piezo1 can promote cancer cell migration and invasion by regulating cell motility–related signaling or remodeling the cytoskeleton. Moreover, in certain cancers, such as esophageal and cervical cancers, Piezo1 can upregulate the expression of mesenchymal markers in tumor tissues, thereby promoting epithelial–mesenchymal transition (EMT) [219, 231]. EMT can disrupt intercellular adhesion, facilitating the detachment of cancer cells from the primary site and enhancing their migratory and invasive capabilities [238]. Next, Piezo1 can inhibit cancer cell apoptosis. An example is in bladder cancer, where Piezo1 overexpression suppresses cancer cells apoptosis and promotes survival via the YAP signaling pathway [221]. Finally, in malignancies such as colorectal cancer, GBM, and hepatocellular carcinoma, Piezo1-promoted tumor angiogenesis remodels the tumor microenvironment, supporting tumor growth and survival [212, 217, 233]. In addition, in myeloid leukemia, a type of liquid tumor, Piezo1 promotes the proliferation and survival of leukemia cells [235]. Notably, the situation in lung cancer presents a distinct contrast. In both small-cell and non–small-cell lung cancers (SCLC and NSCLC), Piezo1 expression in tumor tissues exhibits a marked downregulation trend. Specifically, in SCLC, downregulated Piezo1 reduces cell adhesion, thereby enhancing cancer cell migration and invasion [236]. While in non–small-cell lung cancer, reduced Piezo1 promotes the formation of filopodia in cancer cells, thereby enhancing their migratory capacity [237]. Regrettably, the precise molecular mechanisms underlying Piezo1 downregulation in lung cancer remain to be further elucidated. In summary, except for lung cancer, the enhanced expression and function of Piezo1 in the vast majority of known cancers exert pro-oncogenic effects through a variety of mechanisms.

**Table 1:** Expression and major biological functions of Piezo1 in various cancers

Cancer type	Expression	Major biological function	Reference
Gastric cancer	Upregulated	Promoting cancer cell proliferation, migration, and invasion	[208–210]
Colorectal cancer	Upregulated	Promoting cancer cell migration, tumor angiogenesis, and epithelial–mesenchymal transition	[211–213]
Oral cancer	Upregulated	Promoting cancer cell proliferation	[214]
Hepatocellular carcinoma	Upregulated	Promoting cancer cell proliferation, migration/invasion, tumor angiogenesis and epithelial–mesenchymal transition	[215–218]
Esophageal cancer	Upregulated	Promoting cancer cell proliferation, migration/invasion, anti-apoptosis, and epithelial–mesenchymal transition	[219]
Bladder cancer	Upregulated	Promoting cancer cell proliferation, migration/invasion, and anti-apoptosis	[220, 221]
Prostate cancer	Upregulated	Promoting cancer cell proliferation, migration/invasion, and epithelial–mesenchymal transition	[222, 223]
Breast cancer	Upregulated	Promoting cancer cell migration/invasion, and epithelial–mesenchymal transition	[224–226]
Ovarian cancer	Upregulated	Promoting cancer cell proliferation, migration, and epithelial–mesenchymal transition	[227, 228]
Cervical cancer	Upregulated	Promoting cancer cell migration/invasion, and epithelial–mesenchymal transition	[229–231]
Glioblastoma	Upregulated	Promoting cancer cell migration/invasion, and tumor angiogenesis	[232–234]
Myeloid leukemia	Upregulated	Promoting cancer cell proliferation	[235]
Lung cancer	Downregulated	Promoting cancer cell migration/invasion	[236, 237]

## Targeted therapeutic strategies and challenges for Piezo1

Since the landmark identification of Piezo1, a continuous stream of its pharmacological modulators has been documented. These modulators serve indispensable roles both in fundamental research elucidating the function or mechanism of Piezo1 and as clinical candidates for therapeutic interventions. Herein, we summarized the pharmacological modulators identified to date.

### Pharmacological modulators of Piezo1

#### Piezo1 agonist

Most Piezo1 agonists are specific small molecules that act precisely on defined sites of the Piezo1 protein, modulating its gating activity without affecting Piezo2 or other mechanosensitive channels (Table 2). In 2015, Syeda et al. first identified Yoda1 as an agonist of Piezo1, with median effect concentration (EC_50_) values of 26.6 μM and 17.1 μM for human and mouse Piezo1, respectively [239]. Subsequently, a series of Yoda1-derived analogues, including KC159, KC289 (also known as Yoda2), Yaddle1, and compound 12a (*(R)-1-((5-(5-(((2,6-Dichlorophenyl)methyl-d2)thio)1,3,4-thiadiazol-2-yl)pyrazin-2-yl)amino)propan-2-ol*), were identified, exhibiting improved efficacy, solubility, or stability [240–242]. In 2018, Jedi1 and Jedi2, two milder Piezo1 agonists compared to Yoda1, were identified by Wang et al. through high-throughput screening, with EC_50_ values of about 200 μM and 158 μM in HEK293 cells, respectively [243]. Recently, the Piezo1 agonists CMPD15 and CMPD64 were discovered by Jiang et al. through virtual screening of 8 million compounds using the site-identification by ligand competitive saturation (SILCS), specifically focusing on the Yoda1 binding region [244]. CMPD15 exhibits a potency comparable to that of Yoda1 (EC_50_: 12.9 ± 4.73 µM), whereas CMPD64, identified simultaneously, shows only weak Piezo1 agonistic activity, inducing a modest Ca^2+^ influx only at a concentration of 150 µM [244].

**Table 2:** Pharmacological agonists of Piezo1

Name	Structure	EC_50_	Feature	Activation mechanism	Reference
Yoda1		26.6 μM (hPiezo1)	Specific	Intracellular binding to the transmembrane domain and lowering the activation threshold of Piezo1	[239]
KC159		2.28 μM (hPiezo1)	Specific	Similar to Yoda1	[240]
KC289(Yoda2)		1.14 μM (hPiezo1)	Specific	Similar to Yoda1	[240]
Yaddle1		0.40 μM (hPiezo1)	Specific	Similar to Yoda1	[241]
Compound 12a		2.21 μM (mPiezo1)	Specific	Similar to Yoda1	[242]
Jedi1		~ 200 μM (mPiezo1)	Specific	Extracellular binding to the peripheral blades and amplifying signaling through blade–beam lever-like transduction	[243]
Jedi2		~ 158 μM (mPiezo1)	Specific	Similar to Jedi1	[243]
CMPD15		12.9 µM (mPiezo1)	Specific	Similar to Yoda1	[244]
CMPD64		~ 150 μM (mPiezo1)	Specific	Similar to Yoda1	[244]

#### Piezo1 inhibitors

Piezo1 inhibitors have been identified from various sources, including ions, peptides, fatty acids, and natural products (Table 3). Initially, ruthenium red (RR) is a cationic dye dating back to the 19th century, functioning as a broad-spectrum cation channel inhibitor with non-specific effects on various cation channels [253]. Concurrent with the discovery of the Piezo protein family, Coste et al. demonstrated that RR is a blocker of Piezo1-induced mechanosensitive currents in C2C12 cells (mouse myoblasts), with the half maximal inhibitory concentration (IC_50_) value of 5.4 μM [2, 254]. Like RR, gadolinium (Gd^3+^), a trivalent rare earth metal, non-specifically blocks various mechanosensitive cation channels, including Piezo1 [255]. In terms of potency, 30 μM of Gd^3+^ blocked approximately 84.3% of the mechanically stimulated Piezo1-induced current [2]. Although RR and Gd3 + were discovered and applied early as mechanosensitive channel inhibitors, constituting important tools in the early research of Piezo1, their relatively poor specificity may introduce complexity and interference in the interpretation of experimental results.

**Table 3:** Pharmacological inhibitors of Piezo1

Name	Structure	IC_50_	Feature	Inhibition mechanism	Reference
Ruthenium red		5.4 μM (mPiezo1)	Non-specific	Physically blocking the central pore of Piezo1	[2]
Gd^3+^	-	-	Non-specific	Extracellularly binding to negatively charged membrane components and impeding mechanotransduction	[2]
GsMTx4		~ 2 μM (hPiezo1)	Non-specific	Embedding in the lipid bilayer and reducing mechanical force on the Piezo1 blade domain	[23, 245]
Amyloid β		-	Non-specific	Similar to GsMTx4	[246]
Margaric acid		28.3 μM (mPiezo1)	Non-specific	Embedding in the lipid bilayer and increasing membrane bending rigidity	[30]
Arachidonic acid		-	Non-specific	Accelerating Piezo1 inactivation gating	[30]
Eicosapentaenoic acid		-	Non-specific	Similar to arachidonic acid	[30]
Dooku1		1.49 μM (hPiezo1)	Yoda1 antagonist	Competitive with Yoda1	[247]
Tubeimoside-1		1.11 μM (hPiezo1)	Yoda1 antagonist	Competitive with Yoda1	[248]
Salvianolic acid B		1.37 μM (hPiezo1)	Relatively specific	Competitive with Yoda1 and blocking mechanical force	[249]
Escin		1.74 μM (hPiezo1)	Relatively specific	Competitive with Yoda1 and blocking mechanical force	[250]
Benzbromarone		4.3 μM (hPiezo1)	Drug repurposing	Unclear	[251]
Cannabidiol		0.847 μM (hPiezo1)	Drug repurposing	Unclear	[252]

Among peptide inhibitors, GsMTx4, a 34-amino-acid peptide primarily derived from the venom of Grammostola spiders, is the most widely used Piezo1 inhibitor in preclinical research [256]. In 2011, Bae et al. first reported that GsMTx4 inhibits Piezo1 with a dissociation constant (KD) of ~155 nM in Piezo1-transfected HEK293 cells [23, 245]. Remarkably, its L- and D-enantiomers exhibited similar channel inhibitory activity, supporting that GsMTx4 might act as a gating modifier by altering local membrane tension [257]. Specifically, in an unstressed membrane, GsMTx4 primarily stabilizes on the membrane surface in a shallow binding mode through its six lysine residues. When the membrane is under tension, it moves deeper to alter the distribution of membrane tension, thereby reducing the effective stimuli transmitted to the Piezo1 channel gate and inhibiting channel activity [257]. Similarly, L- and D-enantiomers of amyloid β (Aβ), known for their involvement in Alzheimer’s disease, have been demonstrated to exert comparable inhibitory effects on Piezo1, exhibiting a membrane tension-related inhibitory mechanism similar to that of GsMTx4 [246]. Interestingly, recent research findings have indicated a close association between the accumulation of Aβ peptides and the Piezo1 upregulation in reactive astrocytes and microglia, highlighting the need for further research into Aβ’s regulatory mechanisms on Piezo1 and its therapeutic implications [258–260].

Saturated and polyunsaturated fatty acids both belong to dietary fatty acids and are important components of the plasma membrane, also modulate Piezo1 [261]. In 2019, Romero et al. first identified the margaric acid (MA), a saturated fatty acid, inhibits Piezo1 in Neuro-2a cells (mouse neuroblasts) in a concentration-dependent manner (IC50 = 28.3 ± 3.4 µM) by increasing membrane rigidity and lipid order [30]. Intriguingly, it has negligible effect on Piezo2-induced currents, presumably due to the insensitivity of Piezo2 to membrane rigidity [262]. Moreover, polyunsaturated fatty acids including arachidonic acid (AA) and eicosapentaenoic acid (EPA), have been found to inhibit Piezo1 channels by decreasing membrane rigidity. Of note, EPA and MA exhibit synergistic effects, suggesting that dietary fatty acid intervention may represent a novel strategy to mitigate aberrant Piezo1 activation in human diseases [30].

Recently, an increasing number of phytochemicals have been identified as Piezo1 inhibitors. Most of them share a mechanism similar to that of Dooku1 (a small-molecule Piezo1 inhibitor), competitively and reversibly blocking Yoda1-induced activation of Piezo1 [247]. For instance, tubeimoside-1 (TBMS-1) and salvianolic acid B (SalB), which are extracted from the traditional Chinese medicines *Bolbostemma Rhizoma* and *Salvia miltiorrhiza Bunge*, respectively. They exert concentration-dependent and reversible antagonism competitively binding to the interaction site between Yoda1 and Piezo1 [247–249]. Furthermore, Piezo1 activated by mechanical stimulation can also be effectively inhibited by SalB, although the underlying mechanism warrants further investigation [249]. Additionally, in 2023, Wang et al. found that Yoda1-activated Piezo1-induced Ca^2+^ influx in HUVECs can be inhibited in a concentration-dependent manner by escin (IC50 = 1.74 μM) [250]. Escin is a mixture of triterpenoid saponin extracted from the mature seeds of the *Aesculus hippocastanum* (commonly known as horse chestnut) [263]. Generally, it primarily consists of two components, α-escin and β-escin, with the latter being mainly responsible for its pharmacological activities, including anti-inflammatory, antioxidant, antitumor, antibacterial, and antiviral effects [264–266]. Significantly, escin effectively attenuated the sensitivity of HUVECs to mechanical stimulation, downregulating the expression of inflammatory cytokines like IL-1β and IL-6 that are typically upregulated by Piezo1 under mechanical stress [250]. Currently, escin has been clinically applied, primarily for the treatment of chronic venous insufficiency (CVI) and postoperative edema [267]. Therefore, its pharmacokinetic, pharmacodynamic, and safety profiles are relatively well established.

Notably, drug repurposing has made significant contributions, with several clinically experienced drugs being recently identified as potential Piezo1 modulators. Benzbromarone was originally developed as a potent uricosuric agent and was primarily used for the treatment of hyperuricemia and gout [268]. However, due to reports of severe and even fatal hepatotoxicity, benzbromarone has not received approval from the U.S. Food and Drug Administration (FDA) and has been withdrawn from the market in many countries [269]. In 2024, Liang et al. discovered that benzbromarone directly blocks Piezo1 (IC_50_ = 4.3 ± 0.98 μM), markedly attenuating intracellular calcium overload and hemolysis in HX [251]. Subsequently, in 2026, Liang et al. further reported in a bioRxiv preprint that cannabidiol (CBD), a major non-psychoactive phytocannabinoid that has been approved by the U.S. Food and Drug Administration (FDA) for the treatment of seizures associated with Lennox–Gastaut syndrome, Dravet syndrome, and tuberous sclerosis complex, can also inhibit Piezo1 (IC_50_ = 0.847 ± 0.037 μM) [252, 270]. In addition, CBD has been shown to inhibit Piezo2-mediated mechanosensory signaling and reduces tactile sensitivity in mice [252]. Although the precise molecular mechanisms underlying the inhibition of Piezo1 by benzbromarone and cannabidiol remain unclear, drug repurposing provides an important strategy for the discovery of pharmacological modulators of Piezo1.

### Therapeutic challenges of targeting Piezo1

Although Piezo1 represents a highly promising therapeutic target, the development of treatment strategies targeting Piezo1 remains a formidable challenge. On the one hand, while significant progress has been made in understanding the structure and mechanogating mechanism of Piezo1 channels, most studies have been based on static conformations. The high-resolution static snapshots provided by cryo‑EM are insufficient to capture the full range of continuous conformational transitions, from the resting state to intermediate open, fully open, and inactivated states [28]. The lack of continuous conformational analyses further hinders the precise elucidation of the core Piezo1 mechanogating mechanisms. Moreover, the mechanogating of Piezo1 is strongly influenced by membrane lipid composition, the distribution of membrane tension, and local membrane curvature. Yet, most current studies on Piezo1 structure or mechanogating have been conducted in detergent or artificial lipid systems, which differ from the native cellular membrane environment. This discrepancy introduces uncertainty when correlating in vitro experimental findings with the true physiological state in vivo. Encouragingly, these challenges are being increasingly recognized and progressively addressed. For instance, advanced imaging techniques, such as minimal photon flux microscopy (MINFLUX) and single-particle cryogenic light microscopy (spCryo-LM), together with the application of native cell membrane vesicles, have enabled the dynamic observation of multiple Piezo1 conformations in native cellular membranes [10, 19, 271–273]. Taken together, the incomplete understanding of Piezo1 dynamics conformations and mechanogating mechanisms limits the development of modulatory strategies, including the pharmacological modulators discovery, structural optimization, and efficacy prediction.

On the other hand, because Piezo1 is widely expressed across various tissues and cells to execute diverse physiological functions, any non-specific, systemic activation or inhibition of the channel could result in severe side effects. For instance, systemic administration of the Piezo1 agonist Yoda1 has been associated with risks of hemolysis, disruption of vascular barrier integrity, thrombosis, and potential neuro-cardiovascular disorders [13]. Furthermore, because Piezo1 shares high structural homology with its isoform Piezo2 and functional similarities with other mechanosensitive channels, existing candidate molecules often exhibit significant off-target effects. In particular, there is currently a lack of highly specific Piezo1 inhibitors. Additionally, while the application of targeted delivery systems (e.g., novel nanocarriers, hydrogels-microsphere, or other localized sustained-release biomaterials and scaffolds) could mitigate off-target effects, current technologies still face numerous challenges [274–276]. These include low delivery efficiency, restricted tissue barrier penetration, inadequate therapeutic concentrations at the lesion site, and the necessity for long-term safety evaluations of in vivo degradation products [277]. Collectively, the broad expression and diverse physiological functions of Piezo1, the off-target risks arising from its structural homology, and the limitations of targeted delivery technologies partially account for why no Piezo1-targeted drugs have entered clinical trials to date, as well as representing major challenges for the clinical translation of Piezo1-targeted therapies.

## Future prospect

Currently, the major challenges in targeting Piezo1 underscore the importance of precise targeted therapies. However, conventional regulation methods, such as chemical agonists or inhibitors, suffer from relatively slow diffusion kinetics, insufficient specificity, and a lack of spatiotemporal precision. In light of this, we propose the following future research directions: 1) exploration of adeno-associated virus (AAV)-mediated gene therapy and mRNA therapy in Piezo1 dysregulation diseases; 2) AI-driven discovery of Piezo1-targeted drugs with high affinity and specificity; 3) development of novel drug delivery systems to advance precision medicine.

To address the challenges of tissue selectivity and long-term efficacy that are difficult to overcome with chemical drugs, gene therapy presents a potential solution, particularly for genetic diseases associated with Piezo1 mutations. For example, AAV gene therapy uses recombinant AAV vectors to deliver functional gene copies to target cells, making it suitable for the long-term, low-immunogenic treatment of various genetic diseases [278]. Depending on the specific serotype, AAV vectors exhibit distinct tissue tropism. For instance, AAV8 infects hepatocytes with high efficiency, whereas AAV9 shows a preference for cardiac and skeletal muscles [279, 280]. Recently, in clinical phase 1/2 trials, AAV gene regulatory therapy has shown positive results for Dravet syndrome caused by sodium channel SCN1A defects. This therapy uses the AAV9 vector to deliver an engineered transcription factor specifically targeting the regulatory regions of the patient’s own defective gene [281]. Similarly, using AAV vectors to deliver specific gene editing tools targeting Piezo1 holds promise for correcting Piezo1 mutations-associated genetic diseases such as DHS and GLD. Unlike the sustained expression achieved with AAV, mRNA therapy delivered via lipid nanoparticle (LNP) enables transient and controllable protein expression, offering distinct advantages in acute injury repair or immune modulation [282]. Since mRNA does not enter the nucleus, there is essentially no risk of genomic integration. Furthermore, its relatively short persistence in the body significantly mitigates potential long-term side effects associated with the alteration of Piezo1 function [283]. At present, the mRNA-LNP platform has been extensively validated in COVID-19 vaccines, and its mature manufacturing processes and high purity characteristics support its potential as an ideal tool for Piezo1-targeted therapies [284].

In parallel, the development of novel, high-affinity, and highly specific Piezo1 modulators remains an urgent need. In recent years, the rise of advanced artificial intelligence (AI) technologies has brought new hope for accelerating the discovery of high-affinity Piezo1 modulators. Through integrating AI algorithms with molecular dynamics simulations, it is possible to identify cryptic binding sites in Piezo1 that are exposed only under mechanical strain. Leveraging these sites can guide the discovery of drugs that more closely adhere to the “molecular wedge” mechanism [244]. Meanwhile, AI-driven integrated platforms that combine deep learning, molecular docking, and quantitative structure–activity relationship (QSAR) analysis enable precise evaluation of the structural characteristics and biological activities of compounds, thereby increasing screening accuracy and efficiency [285]. Moreover, the application of AI in predicting the ADMET properties (absorption, distribution, metabolism, excretion, and toxicity) of potential drug candidates will facilitate a reduction in preclinical failure rates and minimize unnecessary cost expenditures [286]. Hence, AI significantly reduces the time and costs associated with traditional high-throughput screening, serving as a powerful tool to facilitate the translation of Piezo1 research from fundamental discovery into therapeutic applications.

In the implementation of strategies targeting Piezo1, the development of novel precision delivery systems complements drug discovery. Ligand modification of LNPs is a feasible strategy to achieve tissue-specific active targeting. In a recent preclinical study, Yan et al. utilized mannose-modified liposomes to specifically target the mannose receptors on the surface of synovial macrophages for the delivery of siRNA, thereby inhibiting Piezo1 expression [287]. Nevertheless, for clinical translation, ligand-modified LNPs still face a series of critical issues that require validation, including ligand stability, human immune response and clearance, as well as the heterogeneity of receptor expression among individuals. Notably, mechanical force-responsive drug delivery systems are intelligent delivery platforms that employ mechanical forces as stimulus signals to control drug transport or release [288]. Such delivery systems may exert exceptional effects at pathological sites where Piezo1 is aberrantly activated due to frequent mechanical stimulation. However, no relevant studies have been reported to date, and this field remains unexplored.

## Conclusion

In conclusion, as a novel mechanosensitive ion channel, the understanding of Piezo1’s structure and mechanogating mechanisms continues to evolve. Its widespread expression in the human body confers diverse biological functions, enabling it to serve as a key driver in the pathogenesis of various human diseases. While this also presents significant challenges for developing Piezo1-targeted therapeutic strategies, the advancement of cutting-edge technologies, including gene therapy, AI-driven drug discovery, and novel targeted delivery systems, offer feasible avenues for clinical translation. Regardless, Piezo1 remains a highly promising therapeutic target, warranting further in-depth exploration.

## Data Availability

Data availability is not applicable to this study as no new data were generated or analyzed in this study.

## Abbreviations

AA: Arachidonic acid
AAV: Adeno-associated virus
Aβ: Amyloid β
ACE2: Angiotensin-converting enzyme 2
AI: Artificial intelligence
AJs: Adherens junctions
AKT: Protein kinase B
APCs: Antigen-presenting cells
AS: Ankylosing spondylitis
ASC: Apoptosis-associated speck-like protein containing a CARD
ATF4: Activating transcription factor 4
ATP: Adenosine triphosphate
BCR: B cell receptor
BKCa: Large-conductance Ca^2+^-activated K^+^
BMDC: Bone marrow-derived dendritic cell
BMSCs: Bone marrow mesenchymal stem cells
BUN: Blood urea nitrogen
CADM1: Cell adhesion molecule 1
CAIA: Collagen antibody-induced arthritis
CALCRL: Calcitonin receptor-like
CaMKII: Calcium/calmodulin-dependent protein kinase II
cAMP: Cyclic adenosine monophosphate
caspase-1: Cysteine-aspartic acid protease 1
CBD: Cannabidiol
CCL2: C C motif ligand 2
CD: Crohn’s disease
C/EBP: CCAAT/enhancer-binding protein
CLR: C-type lectin receptor
CNS: Central nervous system
COVID-19: Coronavirus Disease 2019
cPKC: Classical protein kinase C
CRISPR: Clustered regularly interspaced short palindromic repeats
cryo-EM: Cryo-electron microscopy
CTD: C-terminal domain
CVI: Chronic venous insufficiency
DAI: Disease activity index
DCs: Dendritic cells
DHS: Dehydrated hereditary stomatocytosis
DRG: Dorsal root ganglion
DSS: Dextran sulfate sodium
EAE: Experimental autoimmune encephalomyelitis
EC_50_: Half maximal effective concentration
ECM: Extracellular matrix
ECs: Endothelial cells
EMT: Epithelial–mesenchymal transition
eNOS: Endothelial nitric oxide synthase
EPA: Eicosapentaenoic acid
FAK: Focal adhesion kinase
FDA: U.S. Food and Drug Administration
GBM: Glioblastoma
GD: Graves’ disease
Gd^3+^: Gadolinium
GLD: Generalized lymphatic dysplasia
GLS1: Glutaminase 1
GOF: Gain-of-function
GPR146: G protein-coupled receptor 146
HDFN: Hemolytic disease of the fetus and newborn
HIF-1α: Hypoxia-inducible factor 1-α
HX: Hereditary xerocytosis
IBD: Inflammatory bowel disease
IC_50_: Half maximal inhibitory concentration
ICAM-1: Intercellular adhesion molecule-1
IH: Inner helix
IKCa: Intermediate-conductance Ca^2+^-activated K^+^
IL: Interleukin
ILC: Innate lymphoid cell
KD: Dissociation constant
KLF: Krüppel-like factor
Lcn2: Lipocalin-2
LEC: Lymphatic endothelial cell
LMPHM6: Lymphatic malformation type 6
LNP: Lipid nanoparticle
LOF: Loss-of-function
LPS: Lipopolysaccharide
LN: Lupus nephritis
MA: Margaric acid
MC: Mast cell
mESCs: Mouse embryonic stem cells
MHC: Major histocompatibility complex
MINFLUX: Minimal photon flux microscopy
MS: Multiple sclerosis
MT1-MMP: Membrane-type 1 matrix metalloproteinase
MuSC: Muscle stem cell
NDR1/2: Nuclear Dbf2-related kinase 1/2
NETs: Neutrophil extracellular traps
NFATc1: Nuclear factor of activated T cells 1
NIHF: Nonimmune fetal hydrops
NLRP3: NOD-like receptor thermal protein domain associated protein 3
NO: Nitric oxide
NOX4: NADPH oxidase 4
NSCLC: Non–small-cell lung cancers
NSCs: Neural stem cells
OF: Orbital fibroblast
OH: Outer helix
OLCs: Osteo-chondral lineage cells
OSS: Oscillatory shear stress
p-MLC: Regulating phospho-myosin light chain 2
PC2: Polycystin-2
PECAM1: Platelet endothelial cell adhesion molecule 1
PfEMP-1: Plasmodium falciparum erythrocyte membrane protein 1
PINP: Procollagen type I N-terminal propeptide
PKA: Protein kinase A
PLA2: Phospholipase A2
PLB: Planar lipid bilayer
PMN: Polymorphonuclear leukocyte
PPARγ: Peroxisome proliferator-activated receptor γ
PTGS2: Prostaglandin-endoperoxide synthase 2
QSAR: Quantitative structure–activity relationship
Rac1/2: Ras-related C3 botulinum toxin substrate 1/2
RGC: Retinal ganglion cell
ROS: Reactive oxygen species
RR: Ruthenium red
RVD: Regulatory volume decrease
S1P: Sphingosine-1-phosphate
SalB: Salvianolic acid B
SARS-CoV-2: Severe acute respiratory syndrome coronavirus 2
SCLC: Small-cell lung cancers
SILCS: Site-identification by ligand competitive saturation
SLE: Systemic lupus erythematosus
spCryo-LM: Single-particle cryogenic light microscopy
S-RBD: Spike protein receptor-binding domain
Stoml3: Stomatin-like protein 3
TAO: Thyroid-associated ophthalmopathy
TBMS-1: Tubeimoside-1
TCR: T cell receptor
TED: Thyroid eye disease
TGF-β: Transforming growth factor-β
THBD: Thrombomodulin
THU: Transmembrane helical unit
Th1: T helper 1
TLR4: Toll-like receptor 4
TM helices: Transmembrane helices
TNF-α: Tumor necrosis factor-alpha
Treg: Regulatory T cell
TRPV4: Transient receptor potential vanilloid 4
TTR: Transthyretin
UC: Ulcerative colitis
VCAM-1: Vascular cell adhesion molecule-1
VECs: Vascular endothelial cells
VRAC: Volume-regulated anion channels
VSMC: Vascular smooth muscle cell
WSS: Wall shear stress
YAP: Yes-associated protein
3D: Three-dimensional
